# Amino Acid Substitutions in Positions 385 and 393 of the Hydrophobic Region of VP4 May Be Associated with Rotavirus Attenuation and Cell Culture Adaptation

**DOI:** 10.3390/v12040408

**Published:** 2020-04-07

**Authors:** Yusheng Guo, David E. Wentworth, Karla M. Stucker, Rebecca A. Halpin, Ham Ching Lam, Douglas Marthaler, Linda J. Saif, Anastasia N. Vlasova

**Affiliations:** 1 Food Animal Health Research Program, Ohio Agricultural Research and Development Center, Department of Veterinary Preventive Medicine, The Ohio State University, Wooster, OH 44691, USA; guo.1288@osu.edu; 2 Centers for Disease Control and Prevention, Atlanta, GA 30333, USA; dwentworth@cdc.gov; 3 J. Craig Venter Institute, Rockville, MD 20850, USA; kms36@cornell.edu (K.M.S.); beckyhalpin@gmail.com (R.A.H.); 4 Veterinary Diagnostic Laboratory, University of Minnesota, Saint Paul, MN 55108, USA; lamx0031@umn.edu; 5 Veterinary Diagnostic Laboratory, College of Veterinary Medicine, Kansas State University, Manhattan, KS 66506, USA; marth027@umn.edu

**Keywords:** rotavirus, VP4, attenuation, vaccine, cell culture adaptation

## Abstract

Rotaviruses (RVs) are the leading cause of the acute viral gastroenteritis in young children and livestock animals worldwide. Although live attenuated vaccines have been applied to control RV infection for many years, the underlying mechanisms of RV attenuation following cell culture adaption are unknown. To study these mechanisms at the genomic level, we have sequenced and conducted a comparative analysis of two virulent human (Wa, G1P**[8]** and M, G3P**[8]**) and two virulent porcine (Gottfried, G4P**[6]** and OSU, G5P**[7]**) RV strains maintained in gnotobiotic piglets for 22, 11, 12 and 9 serial passages, respectively, with their attenuated counterparts serially passaged in MA-104 cell cultures for 25, 43, 54 and 43 passages, respectively. We showed that most of the mutations were clustered in the VP4 gene, with a relatively high nonsynonymous substitution rate (81.2%). Moreover, two amino acid substitutions observed in the VP4 gene were conserved between two or more strain pairs. D385N substitution was found in M, Wa and Gottfried strains, and another one, S471H/L was present in Wa and Gottfried strains. Importantly, D385 was reported previously in another study and may be involved in regulation of virus entry. Of interest, although no 385 substitution was found in OSU strains, the attenuated OSU strain contained a unique D393H substitution within the same VP4 hydrophobic domain. Collectively, our data suggest that the VP4 hydrophobic region may play an important role in RV attenuation and aa385 and aa393 may represent potential targets for RV vaccine development using reverse genetics and site-specific mutagenesis.

## 1. Introduction

Rotaviruses (RVs), members of Reoviridae family, are the leading cause of acute viral gastroenteritis in children under 5 years of age and in young livestock worldwide [1,2]. RVs cause approximately $1 billion in annual costs due to hospitalizations in the United States [3,4,5]. In addition, RVs are responsible for 7–50% mortality in piglets, resulting in major economic losses to the pork industry.

The classical approach to virus attenuation relies on serial passaging of the virulent wild-type viruses in susceptible cells in vitro until partial or complete attenuation is achieved [6]. For many years, it has been the most common strategy to generate many live attenuated vaccines. In the case of RV, Rotarix^®^ (GlaxoSmithKline, GSK) was developed using this approach. Although cell culture adaptation has been widely used to attenuate virus strains and for vaccine development, the underlying mechanisms and functions of the individual RV genes remain largely undefined. Hoshino et al. reported that VP3, VP4, VP7 and the NSP4 genes played important roles in the pathogenesis of RV in gnotobiotic piglets [7]. Chang and colleagues compared sequences of the NSP4 genes of virulent and attenuated group A RV Wa and M (RVA) and group C RV Cowden strains (RVC). They found several amino acid (aa) substitutions in the Wa and Cowden strains [8]; however, no mutations common between the different strain pairs were reported, suggesting that RVA and RVC attenuation may not be directly related to difference in the NSP4 gene sequence. Other researchers have analyzed sequences of murine (EB) and human (Wa, DC3695 and DC5685) RVA strains, and also found some nonsynonymous mutations in the VP4, VP7 and NSP4 genes [9,10]. Interestingly, these mutations were cell line specific, suggesting that viral attenuation and cell culture adaptation mechanisms may be redundant. Among those mutations, only the substitution at amino acid 385 in the VP4 of human RV (HRV) was common between the attenuated pairs of Wa, DC3695 and DC5685 [10]. Further, aa37 (V→A) substitution in the NSP4 gene of EB strain was confirmed to be a key residue involved in the virus pathogenicity following experiments with recombinant NSP4 proteins conducted in mice [9]. Although some polymorphic sites have been reported, little is known about whether these mutations are conserved across different HRV and animal RV (ARV) strains.

To explore which gene segments and specific mutations were associated with RV attenuation, we sequenced the complete genomes of two virulent HRV strains: M (G3P**[8]**, M-V) and Wa (G1P**[8]**, Wa-V), and two virulent porcine RV (PRV) strains: Gottfried strain (G4P**[6]**, Got-V) and OSU strain (G5P**[7]**, OSU-V) and their corresponding attenuated counterparts. These RVs were serially passaged in gnotobiotic pigs to maintain virulent phenotype, or in African green monkey cell line MA104 to achieve attenuation.

## 2. Materials and Methods

### 2.1. Rotavirus Strains

Pairs of virulent and cell culture adapted (attenuated) human/animal group A RVs included: human G1P**[8]** Wa strain, human G3P**[8]** M strain, porcine G4P**[6]** Gottfried strain and porcine G5P**[7]** OSU strain. The virulent strains were serially passaged in gnotobiotic pigs for 9 (Gottfried), 12 (OSU), 11 (M) and 22 (Wa) passages as described [11,12], and their attenuated counterparts were serially passaged in an MA-104 cell culture for 54 (Gottfried), 43 (OSU), 43 (M) and 25 (Wa) consecutive passages, as described [13]. The attenuation status of the cell culture adapted variants was confirmed in Gn piglets, as described [11,12]. Attenuated RVs did not induce any diarrhea in Gn piglets at any dose given including the highest, while fecal virus shedding and seroconversion in the piglets were confirmed.

### 2.2. Complete Genomic Sequencing of Human/Animal Group A Rotavirus Strains

RNA extraction and purification for Illumina^®^ (complete genome) sequencing. Complete genome sequencing of porcine and human rotaviruses was conducted as previously described [14]. Briefly, stool from 4 samples available in sufficient quantities was resuspended in 10 vol. of phosphate-buffered saline (PBS) and vigorously vortexed for 5 min. The supernatant was collected after centrifugation (5 min, 15,000 × *g*) and filtered through a 0.22 µm filter to remove eukaryotic cell-sized particles. For cell culture adapted RV strains, cell culture supernatants were collected after centrifugation (5 min, 15,000 × *g*) and filtered as above. The filtrates were treated with DNase and RNase to digest unprotected nucleic acid at 37 °C for 90 min. Viral RNA was then extracted using the RNeasy kit (Qiagen), following the manufacturer’s instructions. The concentration and purity of extracted RNA were measured with a spectrophotometer (ND-1000, Nanodrop Technologies). RNA extracts that presented 260/280 and 260/230 purity indices equal to or greater than 2.0 were selected. Subsequently, total RNA was used in library construction using the TruSeq RNA Sample Preparation kit v2 (Illumina^®^, USA). The final size and concentration of each library was estimated using a Bioanalyzer (Agilent, Santa Clara, CA, USA) and the Qubit (Invitrogen, Carlsbad, CA, USA), respectively. Ten nM library pools were prepared by mixing the 8 libraries to achieve an equal molar concentration of each. Pooled libraries were sequenced by the MiSeq (Illumina^®^) platform using sequenced runs of 2 × 150 paired end reads. There were >2–3 × 10^5^ reads/genome, with >50% RV reads and <50% non-RV/host cell sequences. The coverage was >99.9–100% for 5 genomes, and it was 91.6% for attenuated OSU, 94.5% for virulent M and 99.8% for attenuated Gottfried strains. To close the gaps between assembled contigs of the latter three sequences, strain specific primers (Table 1) were designed, RT-PCR was performed, and amplicons were sequenced. All RVA sequences were deposited into GenBank. The GenBank accession numbers are shown in supplemental Table S1.

### 2.3. Nucleotide and aa Sequences Analyze and Comparison

The nucleotide sequences were trimmed and assembled manually by using the DNASTAR program (SeqMan NGen®. Version 7.0. DNASTAR. Madison, WI, USA). Analysis of the nucleotide and the deduced amino acid sequences, and dN/dS ratios was performed directly using the MEGAX software [15] VP4 alignments were constructed in MEGAX, and the Figures were generated using online tool ENDscript 2.X [16].

## 3. Results

### 3.1. Summary of the Total Nucleotide and aa Changes of Four RV Strains

Comparative analysis of the virulent and attenuated strains is summarized in Table 2. Overall, the VP4 gene contained the most nucleotide and amino acid substitutions, followed by VP7, NSP5 and VP3. Notably, VP4 was the only gene that was modified after cell culture adaptation and attenuation of all 4 strains, which indicates that it may play an important role in RV attenuation. Additionally, VP4 was the only gene segment that had mutations following cell culture adaptation and attenuation of the OSU strain, while no mutations were detected in any other OSU gene segments. In addition, nucleotide changes in the VP7 gene were observed only in HRV (M and Wa) but not in PRV strains (Gottfried and OSU). In contrast, NSP5, VP3 and NSP3 showed mutations in both cell cultures adapted attenuated human and porcine strains. Mutations in the NSP1, NSP4, VP1, VP2 and VP3 were all strain specific, and we did not observe any changes in the NSP2 of any of the 4 strains. With regard to nonsynonymous substitution (resulting in different amino acids), VP4 had the most aa substitutions (27), resulting in a high nonsynonymous substitution rate of 81.8%. The nonsynonymous substitution rate in VP7, NSP4, NSP5, VP1, VP6 and NSP3 genes was 100%, however, the total numbers of aa substitutions (5) were substantially lower in these proteins than in VP4. It should be mentioned that NSP1 and NSP2 had the lowest nonsynonymous and overall substitution rates, with only 1 and 0 aa substitutions, respectively. So, the importance of the aa mutations in NSP1 gene should be evaluated further. Additionally, there were no changes in the 5′- ad 3′ non-coding terminal regions (NCR) of the attenuated RVs, with the exception of one nucleotide substitution (G2518A) in the 3′ NCR of the VP3 gene of attenuated M strain (See in Table 3B).

### 3.2. Mutations Detected After Serial Passaging The RVA Strains in MA104 Cell Culture

**Figure 1:**
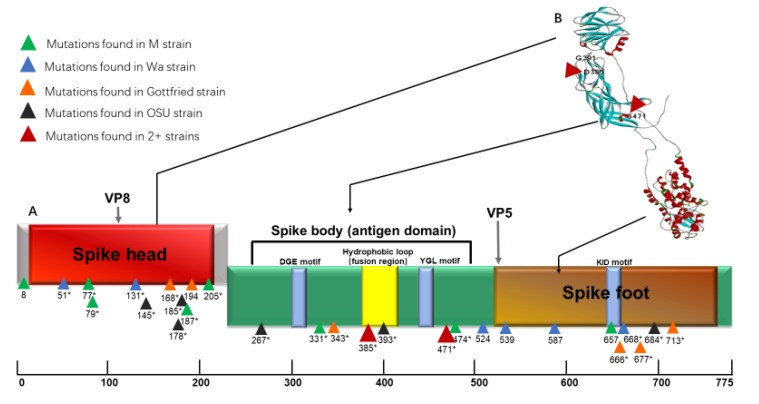
Structure of rotavirus VP4 spike protein. Linear schematic organization (**A**) and 3D structure (**B**) including VP8* and VP5* sub-units, spike head, spike body and spike foot are shown. Mutations localized in each domain are shown as triangles and numbers under these triangles denote amino acid position with asterisks indicating non-synonymous mutations. The 3D structure was generated by using online protein modeling tool Phyre2 [17].

All nucleotide and amino acid substitutions detected in each of the 10 segments (NSP2 was not included since no substitution was detected) of the four attenuated strains are summarized in Table 3 (See VP4 gene as Table 3A, and the rest 9 genes as Table 3B). One aa substitution site in the VP4 gene, D385N, was identified in the three RVA strain pairs, but not in OSU that, however, had a unique substitution, D393H, which was not found in any other strains and was located within the same hydrophobic loop as the D385N (Figure 1). After nucleotide and protein sequence alignment, we found that the original nt in position 1153 (aa385) in virulent OSU strain was “Y”, however, the other three virulent strains had “G” in this position substituted with A in their attenuated counterparts and resulting in the D385N aa substitution (see supplementary Figures S1–S4 for the pairwise VP4 alignment information for Wa, M, Gottfried and OSU, respectively.) In addition, aa S in the position 471 was replaced in both Wa and Gottfried strains (Figure 1). Two nt substitutions (1411 T → C and 1412 C → A) have resulted in the S471H substitution in the Wa strain; whereas, one single nt mutation (1412 C to T) lead to the S471L substitution in the Gottfried strain. Interestingly, a cluster of synonymous mutations was found between positions 500–660 (Figure 1; Table 3). Analysis of the dN/dS ratio for this region of M and Wa strains suggested that this region evolves under a strong negative selective pressure (Supplementary Table S2). This indicates that nonsynonymous mutations in this region may be lethal for HRVs. No common nt or aa polymorphic sites were found in genes other than VP4.

## 4. Discussion

Although cell culture adaptation has been a classical approach for RV vaccine development, the underlying mechanisms are not well understood. In this study, we conducted sequencing and comparative analysis of 2 HRV and 2 PRV strains serially passaged in cell culture and attenuated in Gn pigs [11,12,13] vs. their virulent counterparts. After sequencing and comparing the genomes of the wild-type (virulent) and cell culture adapted attenuated strains, we found that VP4 was the only gene that had nt mutations in all four strains, with a relatively high nonsynonymous substitution rate (81.8%). Moreover, mutations observed in the VP4 gene were more numerous than in all other genes. For example, 6.6- and 11-times more nt mutations (2.9- and 3.3-fold increases after correction by the gene length) were observed in the VP4 gene compared to VP7 and NSP4, respectively. Similar studies have been performed using either human G1P**[8]** [9,18,19,20,21] or murine G16P[16] strains [10], with the VP4 gene mutations consistently identified irrespective of the host species origin. Thus, similar to findings by others, our data suggest that VP4 may be the key gene controlling RVAs adaptation to cell culture.

As one of the outer RV capsid proteins, VP4 plays an important role in virus entry because mature RVs show infectivity only after trypsin cleavage of the VP4 into VP8* and VP5* [22]. Here, we found two common substitution sites (detected in at least two strains) in the “body” region of VP5* (aa 248–490) [23]. One is the D385N, which we found in the VP4 gene of the M, Wa and Gottfried cell culture-adapted attenuated strains, but not in the OSU VP4. However, the VP4 gene of the cell culture-adapted attenuated OSU strain has a unique substitution, D393H, that also falls within the putative cell fusion domain. Notably, D in the positions 385 and 393 are the only negatively charged amino acids in the VP5 hydrophobic loop. So, these D→N and D→H substitutions represent radical changes from a negatively charged (aspartate) to an uncharged/positively charged (asparagine/histidine) amino acid, which are more than likely to result in a conformational change of this epitope (which is responsible for membrane penetration and is targeted by neutralizing antibodies). Interestingly, although D385N or a similar substitution was observed frequently in similar studies [9,10,19,21], the exact function of this site and the effects of the respective substitution remains unclear. It has been reported that 385D was highly conserved in the wild-type P**[8]** RVs, but less conserved in the tissue-culture-attenuated RVs [10], indicating that this site is under a strong selective pressure during cell culture adaptation. In addition, several publications indicated that aa 385 was located at the fusion domain of VP4 (aa 384–404), and thus may have an effect on this region conformation [24,25]. The fact that D385N substitution results in conformational re-arrangements has been reported previously numerous times [26,27]. So, one study identified amino acids 385–392 as a conformational C2 epitope (target for neutralizing antibodies), and selection with MAbs targeting the C2 epitope, which resulted in generation of escape mutants carrying the same D385N substitution [28]. Similarly, substitution of the D residue in the position 393 results in conformational changes and affects the VP5 reactivity with MAbs [29]. Another interesting finding was that the mutation in the VP5* hydrophobic loops (V391D) decreased the in vitro RV infectivity 10,000-fold, as compared to viral particles recoated with the wild-type VP4 [23]. Since D385 is located very close to V391 (Figure 1B), it is reasonable to speculate that the D385N mutation may change the conformation of the hydrophobic loops in the VP5 and lead to decreased intestinal replication of the of cell culture-adapted attenuated RV strains. Further studies are required to explore the function of D385 by using reassortant viruses or recently developed RV reverse genetics system [30]. Further, although no mutation was found in OSU strains at the position 385, the D393H substitution in the attenuated counterpart may indicate that the same hydrophobic region was altered during cell culture adaptation and attenuation.

Another conserved mutation identified in the cell culture adapted attenuated strain found in this study, the S471 substitution (in which Ser was substituted by His (Wa strain) or Leu (Gottfried strain)) was also present in the HRV G**[1]**P**[8]** DC3695 (S471F) strain in the Tsugawa et al. study [10]. However, this site was not discussed in their article because it only occurred in one of the three cell culture adapted strains that they studied, and this mutation was not conserved in the rest of the strains. In our study, S471 was substituted in both the HRV Wa strain and the PRV Gottfried strains with different aa (His in Wa or Leu in Gottfried). Although S471 was substituted in different cell culture-adapted attenuated strains, no common aa substitution has been found. Thus, it is hard to draw a conclusion about the importance of this site in the process of RVA attenuation. Notably, substitutions in several aa positions in the VP4, including 51, 331,343 and 474, were only identified in one of the strains in our study. Nonetheless, these sites have been previously reported in other studies [10,19,21]. Further studies are warranted of the functional importance of these sites using the recently available entirely plasmid-based reverse genetics system for RVs [30].

VP8* domain of VP4 plays an important role in the initial attachment of RV and can differentially bind to carbohydrates such as sialic acid and histo-blood Group Antigens (HBGAs) [31,32,33,34]. In this study, we found a cluster of mutations (aa position 145 to 205, Figure 1) that fall into the previous identified carbohydrates binding region. Among these aa substitution sites, aa 185 and 187 were indicated to form glycan binding pocket in previous studies. So, T185 of a P**[19]** RV are associated with the hydrogen bond formation with mucin core2 [34] and K187, which has been reported to be a part of the sialoside binding core of rhesus RV. Although the importance of polymorphisms in sites other than 185 and 187 remains unclear, the close proximity of these substitutions to the carbohydrate binding region may result in an altered binding of VP8* following the cell culture adaption and attenuation process.

Overall, in the present study, three aa substitutions, D385N, D393H and S471H/L, have occurred alone or in combination after each of the four virulent RV strains were cell culture-adapted and developed an attenuated phenotype [11,12,13]. Thus, our results identify potential VP4 sites that may play a central role in RVA attenuation. Thus, these findings provide further mechanistic evidence of the involvement of VP4 in RVA attenuation, and identify critical epitopes for the development of attenuated vaccines using site-specific mutagenesis.

## Figures and Tables

**Table 1:** Primers used to close sequence gaps.

Primer Name	Sequence (5′→3′)
HRV-M-NSP4-F1	GCGTGCGGAAAGATGGATAAG
HRV-M-NSP4-R352	CAATCATCTCCAGCTGACGTC
HRV-M-NSP4-F258	GGCTGGATATAAAGAGCAGGTTA
HRV-M-NSP4-R697	ATTAACGTCCAACACTCGCTG
HRV-M-VP7-F1	CGTTTGGCTAGCGGTTAGCT
HRV-M-VP7-F309	TGATAACTCATGGAAGGATACAC
HRV-M-VP7-R664	TTGTATCAGTAGTTAGACACCC
HRV-M-VP7-R1023	TAACCTAAGCTATATCTATACTCTG
HRV-M-VP7-FOUT840	AACAGCTGATCCAACGACAG
HRV-M-VP7-ROUT178	GTGCATCAAGGAGTGGTGAC
PRV-OSU-VP4-F730	GTGCACACAAGAGCTCAAGTTA
PRV-OSU-VP4-F1067	GGGATGATTCACAAGCATTCAG
PRV-OSU-VP4-R1151	CTACCACCAGTACACGTTACTG
PRV-OSU-VP4-F1466	CTGTGAGGCAAGATCTAGAGAG
PRV-OSU-VP4-R1514	AACTCATCTCGTAGCTCTCCC
PRV-OSU-VP4-F1772	GGACGGAAGTGTCAAATTCGATC
PRV-OSU-VP4-R1867	CTTTCAATCGTAACCGCTTAGC
PRV-OSU-VP4-F1969	CCGGAAATAGTTACTGAAGCTTC
PRV-OSU-VP4-R2095	CGAATGTGTCTACACGATATGCG
PRV-OSU-VP4-R2336	GGTCACAACTACTTACAGTCTAC

Table summarized the primers that designed and used for filling the sequencing gap. Primers are designed using MEGA5 and quality of primers are checked by using online tools: Oligo Calc: Oligo nucleotide Properties Calculator.

**Table 2:** Summary of nucleotide and deduced amino acid substitutions in the 11 RVA gene segments.

	Nucleotide/Amino Acid Substitutions										
Strain	VP4	VP7	NSP5	VP3	NSP1	NSP4	VP1	VP2	VP6	NSP3	NSP2
M	10/8	1/1	0/0	2/2	0/0	0/0	0/0	0/0	3/3	0/0	0/0
Wa	9/6	4/4	2/2	0/0	0/0	3/3	2/2	0/0	0/0	1/1	0/0
OSU	8/7	0/0	0/0	0/0	0/0	0/0	0/0	0/0	0/0	0/0	0/0
Gottfried	6/6	0/0	1/1	2/1	3/1	0/0	0/0	3/2	0/0	1/1	0/0
Total	33/27	5/5	3/3	4/3	3/1	3/3	2/2	3/2	3/3	2/2	0/0
Nonsynonymous Substitution rate (%) ^a^	81.8	100	100	75	33.3	100	100	66.7	100	100	0

^a.^ Nonsynonymous substitution rate (%) was calculated using the following formula: (amino acid substitution number/nucleotide substitution number) *100%.

**Table 3:** Summary of all mutations identified in the 10 gene segments of the cell culture adapted attenuated RVA strains.

**A**					
	**nt**				
**Gene**	**aa**	**M**	**Wa**	**OSU**	**Gottfried**
VP4	24	A→G			
	8	Gln			
	152		G→T		
	51		Gly→Val		
	230	C→T			
	77	Pro→Leu			
	235	A→G			
	79	Asn→Gly			
	236	A→G			
	79	Asn→Gly			
	391		C→A		
	131		Arg→Ser		
	434			A→C	
	145			Lys→Thr	
	503				A→C
	168				Lys→Thr
	532			G→A	
	178			Asp→Asn	
	554			T→C	
	185			Ile→Thr	
	559	G→A			
	187	Gly→Ser			
	582				T→C
	194				Pro
	613	T→C			
	205	Tyr→His			
	800			A→G	
	267			Tyr→Cys	
	992	C→T			
	331	Ser→Phe			
	1027				G→T
	343				Val→Leu
	1153	G→A	G→A		G→A
	385	Asp→Asn	Asp→Asn		Asp→Asn
	1177			G→C	
	393			Asp→His	
	1411		T→C		
	471		Ser→His		
	1412		C→A		C→T
	471		Ser→His		Ser→Leu
	1420	C→T			
	474	Pro→Ser			
	1572 (!)		A→T		
	524 (!)		Leu		
	1617		A→G		
	539		Leu		
	1761		T→C		
	587		Asp		
	1971	C→T			
	657	Asp			
	1997				T→C
	666				Phe→Ser
	2003		C→T		
	668		Pro→Leu		
	2029				G→A
	677				Asp→Asn
	2051			C→T	
	684			Asp→His	
	2137				G→A
	713				Asp→Asn
**B**					
	**nt**				
**Gene**	**aa**	**M**	**Wa**	**OSU**	**Gottfried**
NSP1	1038				T→C
	346				Tyr
	1074				G→A
	358				Leu
	1133	C→T			
	378	Thr→Met			
NSP3	247				G→T
	83				Ala→Ser
	859		A→G		
	287		Ile→Val		
NSP4	131		T→C		
	50		Val→Ala		
	59		T→C		
	20		Leu→Ser		
	113		C→T		
	38		Pro→Leu		
NSP5	91				T→C
	31				Phe→Leu
	143		A→G		
	48		Asn→Ser		
	209		G→A		
	70		Arg→Gln		
VP1	1936		G→A		
	646		Ala→Thr		
	3023		G→A		
	1008		Arg→Gln		
VP2	1290				G→A
	430				Leu
	1949				C→T
	650				Ser→Leu
	2623				C→T
	875				Pro→Ser
VP3	282	G→A			
	94	Met→Ile			
	794				A→G
	265				Leu
	2202				G→A
	734				Met→Ile
	2518	G→A			
		Glu→Lys			
VP6	169	A→T			
	57	Ile→Val			
	475	C→T			
	159	Leu→Phe			
	1063	G→A			
	355	Ala→Thr			
VP7	223	A→C			
	75	Thr→Leu			
	224	C→T			
	75	Thr→Leu			
	293		C→T		
	98		Ala→Val		
	472		G→A		
	158		Glu→Lys		
	823		T→C		
	275		Ser→Pro		
	869		C→T		
	290		Thr→Ile		

Table 3 summarizes all the substitutions identified in the four RVA pairs. “nt” and “aa” indicate nucleotide and amino acid position, respectively. Mutations that are observed in at least two strains are shown in red and bold. Synonymous nt mutations (that did not lead to aa substitution) are shown in blue. (**A**) substitutions identified in VP4 gene; (**B**) substitutions identified in the rest genes.

## Supplementary Materials

The following are available online at https://www.mdpi.com/1999-4915/12/4/408/s1, Figure S1: Amino acid alignment of VP4 gene in Wa strain, Figure S2: Amino acid alignment of VP4 gene in M strain, Figure S3: Amino acid alignment of VP4 gene in Gottfried strain, Figure S4: Amino acid alignment of VP4 gene in OSU strain. Table S1: NCBI accession number of all gene segments, Table S2: dN/dS ratio analyze per site in the region 500-660 of VP4 gene in Wa and M strains.

## References

[1] Tate J.E., Burton A.H., Boschi-Pinto C., Parashar U.D., World Health Organization-Coordinated Global Rotavirus Surveillance N. (2016). Global, Regional, and National Estimates of Rotavirus Mortality in Children <5 Years of Age, 2000–2013. Clin. Infect. Dis..

[2] Vlasova A.N., Amimo J.O., Saif L.J. (2017). Porcine Rotaviruses: Epidemiology, Immune Responses and Control Strategies. Viruses.

[3] Parashar U.D., Hummelman E.G., Bresee J.S., Miller M.A., Glass R.I. (2003). Global illness and deaths caused by rotavirus disease in children. Emerg. Infect. Dis..

[4] Parashar U.D., Gibson C.J., Bresee J.S., Glass R.I. (2006). Rotavirus and severe childhood diarrhea. Emerg. Infect. Dis..

[5] Payne D.C., Staat M.A., Edwards K.M., Szilagyi P.G., Weinberg G.A., Hall C.B., Chappell J., Curns A.T., Wikswo M., Tate J.E. (2011). Direct and indirect effects of rotavirus vaccination upon childhood hospitalizations in 3 US Counties, 2006–2009. Clin. Infect. Dis..

[6] Hanley K.A. (2011). The double-edged sword: How evolution can make or break a live-attenuated virus vaccine. Evolution.

[7] Hoshino Y., Saif L.J., Kang S.Y., Sereno M.M., Chen W.K., Kapikian A.Z. (1995). Identification of group A rotavirus genes associated with virulence of a porcine rotavirus and host range restriction of a human rotavirus in the gnotobiotic piglet model. Virology.

[8] Chang K.O., Kim Y.J., Saif L.J. (1999). Comparisons of nucleotide and deduced amino acid sequences of NSP4 genes of virulent and attenuated pairs of group A and C rotaviruses. Virus Genes.

[9] Tsugawa T., Tatsumi M., Tsutsumi H. (2014). Virulence-associated genome mutations of murine rotavirus identified by alternating serial passages in mice and cell cultures. J. Virol..

[10] Tsugawa T., Tsutsumi H. (2016). Genomic changes detected after serial passages in cell culture of virulent human G1P[8] rotaviruses. Infect. Genet. Evol..

[11] Ward L.A., Rosen B.I., Yuan L., Saif L.J. (1996). Pathogenesis of an attenuated and a virulent strain of group A human rotavirus in neonatal gnotobiotic pigs. J. Gen. Virol..

[12] Bohl E.H., Theil K.W., Saif L.J. (1984). Isolation and serotyping of porcine rotaviruses and antigenic comparison with other rotaviruses. J. Clin. Microbiol..

[13] Saif L., Yuan L., Ward L., To T. (1997). Comparative studies of the pathogenesis, antibody immune responses, and homologous protection to porcine and human rotaviruses in gnotobiotic piglets. Adv. Exp. Med. Biol..

[14] Nyaga M.M., Jere K.C., Esona M.D., Seheri M.L., Stucker K.M., Halpin R.A., Akopov A., Stockwell T.B., Peenze I., Diop A. (2015). Whole genome detection of rotavirus mixed infections in human, porcine and bovine samples co-infected with various rotavirus strains collected from sub-Saharan Africa. Infect. Genet. Evol..

[15] Kumar S., Stecher G., Li M., Knyaz C., Tamura K. (2018). MEGA X: Molecular Evolutionary Genetics Analysis across Computing Platforms. Mol. Biol. Evol..

[16] Robert X., Gouet P. (2014). Deciphering key features in protein structures with the new ENDscript server. Nucleic Acids Res..

[17] Kelley L.A., Mezulis S., Yates C.M., Wass M.N., Sternberg M.J. (2015). The Phyre2 web portal for protein modeling, prediction and analysis. Nat. Protoc..

[18] Arora R., Dhale G.S., Patil P.R., Chitambar S.D. (2011). Sequence analysis of VP4 genes of wild type and culture adapted human rotavirus G1P[8] strains. Asian Pac. J. Trop. Med..

[19] Esona M.D., Foytich K., Wang Y., Shin G., Wei G., Gentsch J.R., Glass R.I., Jiang B. (2010). Molecular characterization of human rotavirus vaccine strain CDC-9 during sequential passages in vero cells. Hum. Vaccin..

[20] Kitamoto N., Mattion N.M., Estes M.K. (1993). Alterations in the sequence of the gene 4 from a human rotavirus after multiple passages in HepG2 liver cells. Arch. Virol..

[21] Ward R.L., Kirkwood C.D., Sander D.S., Smith V.E., Shao M., Bean J.A., Sack D.A., Bernstein D.I. (2006). Reductions in Cross-Neutralizing Antibody Responses in Infants after Attenuation of the Human Rotavirus Vaccine Candidate 89–12. J. Infect. Dis..

[22] Estes M.K., Graham D.Y., Mason B.B. (1981). Proteolytic enhancement of rotavirus infectivity: molecular mechanisms. J. Virol..

[23] Kim I.S., Trask S.D., Babyonyshev M., Dormitzer P.R., Harrison S.C. (2010). Effect of mutations in VP5 hydrophobic loops on rotavirus cell entry. J. Virol..

[24] Estes M., Knipe D.M., Howley P.M., Griffin D.E. (2001). Rotaviruses and their replication. Fields Virology.

[25] López S., Arias C.F. (2004). Multistep entry of rotavirus into cells: A Versaillesque dance. Trends Microbiol..

[26] Dormitzer P.R., Nason E.B., Venkataram Prasad B.V., Harrison S.C. (2004). Structural rearrangements in the membrane penetration protein of a non-enveloped virus. Nature.

[27] Roy S., Esona M.D., Kirkness E.F., Akopov A., McAllen J.K., Wikswo M.E., Cortese M.M., Payne D.C., Parashar U.D., Gentsch J.R. (2014). Comparative genomic analysis of genogroup 1 (Wa-like) rotaviruses circulating in the USA, 2006–2009. Infect. Genet. Evol..

[28] Kobayashi N., Taniguchi K., Urasawa S. (1990). Identification of operationally overlapping and independent cross-reactive neutralization regions on human rotavirus VP4. J. Gen. Virol..

[29] Isegawa Y., Nakagomi O., Nakagomi T., Ueda S. (1992). A VP4 sequence highly conserved in human rotavirus strain AU-1 and feline rotavirus strain FRV-1. J. Gen. Virol..

[30] Kanai Y., Komoto S., Kawagishi T., Nouda R., Nagasawa N., Onishi M., Matsuura Y., Taniguchi K., Kobayashi T. (2017). Entirely plasmid-based reverse genetics system for rotaviruses. Proc. Natl. Acad. Sci. USA.

[31] Dormitzer P.R., Sun Z.-Y.J., Wagner G., Harrison S.C. (2002). The rhesus rotavirus VP4 sialic acid binding domain has a galectin fold with a novel carbohydrate binding site. EMBO J..

[32] Hu L., Crawford S.E., Czako R., Cortes-Penfield N.W., Smith D.F., Le Pendu J., Estes M.K., Prasad B.V. (2012). Cell attachment protein VP8* of a human rotavirus specifically interacts with A-type histo-blood group antigen. Nature.

[33] Liu Y., Huang P., Tan M., Liu Y., Biesiada J., Meller J., Castello A.A., Jiang B., Jiang X. (2012). Rotavirus VP8*: phylogeny, host range, and interaction with histo-blood group antigens. J. Virol..

[34] Liu Y., Xu S., Woodruff A.L., Xia M., Tan M., Kennedy M.A., Jiang X. (2017). Structural basis of glycan specificity of P[19] VP8*: Implications for rotavirus zoonosis and evolution. PLoS Pathog..

