# Supplementary material for: Amino Acid Substitutions in Positions 385 and 393 of the Hydrophobic Region of VP4 May Be Associated with Rotavirus Attenuation and Cell Culture Adaptation
Source: Viruses. 2020 Apr 7;12(4):408. doi: 10.3390/v12040408 (PMC7232350; doi:10.3390/v12040408)
Supplement: Supplementary file 1 [file viruses-12-00408-s001.pdf]

viruses

Supplementary Materials: Amino Acid Substitutions in Positions 385 and 393 of the Hydrophobic Region of VP4 May Be Associated with Rotavirus Attenuation and Cell Culture Adaptation

Yusheng Guo, David E. Wentworth, Karla M. Stucker, Rebecca A. Halpin, Ham Ching Lam, Douglas Marthaler, Linda J. Saif and Anastasia N. Vlasova

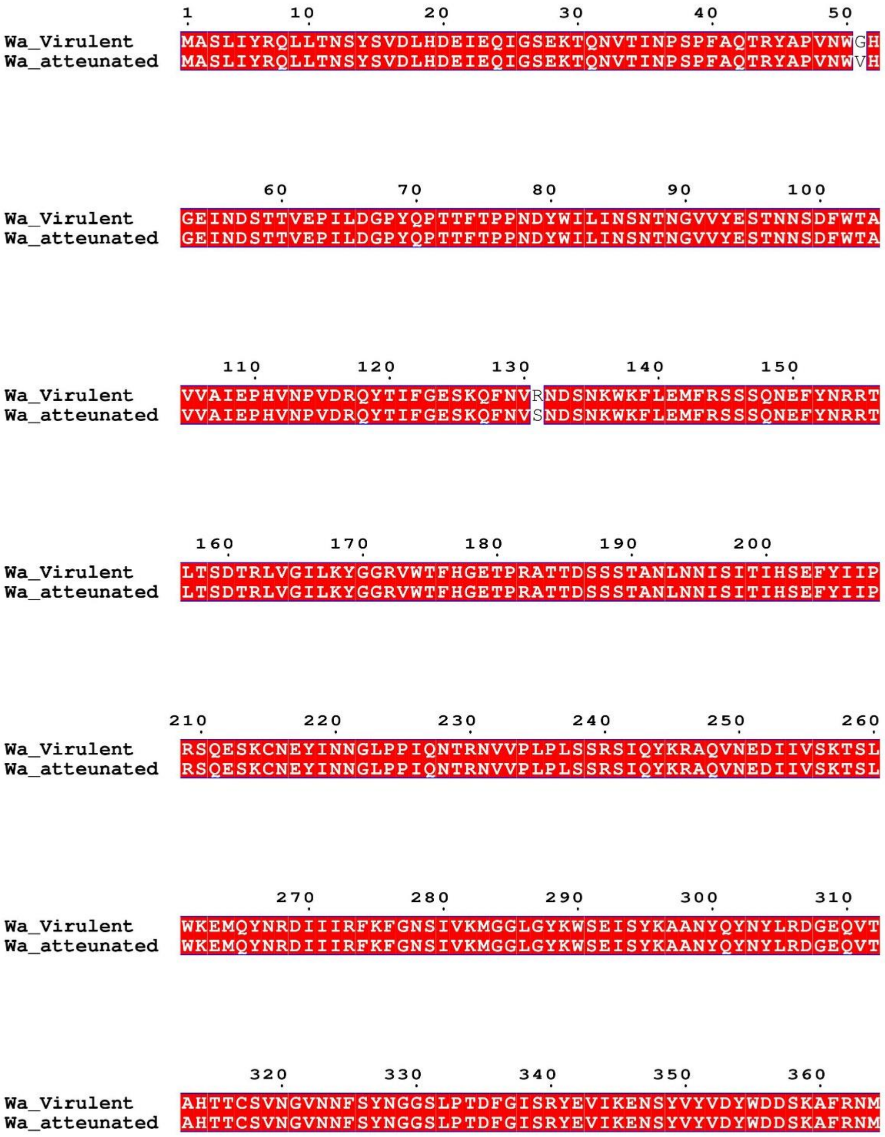

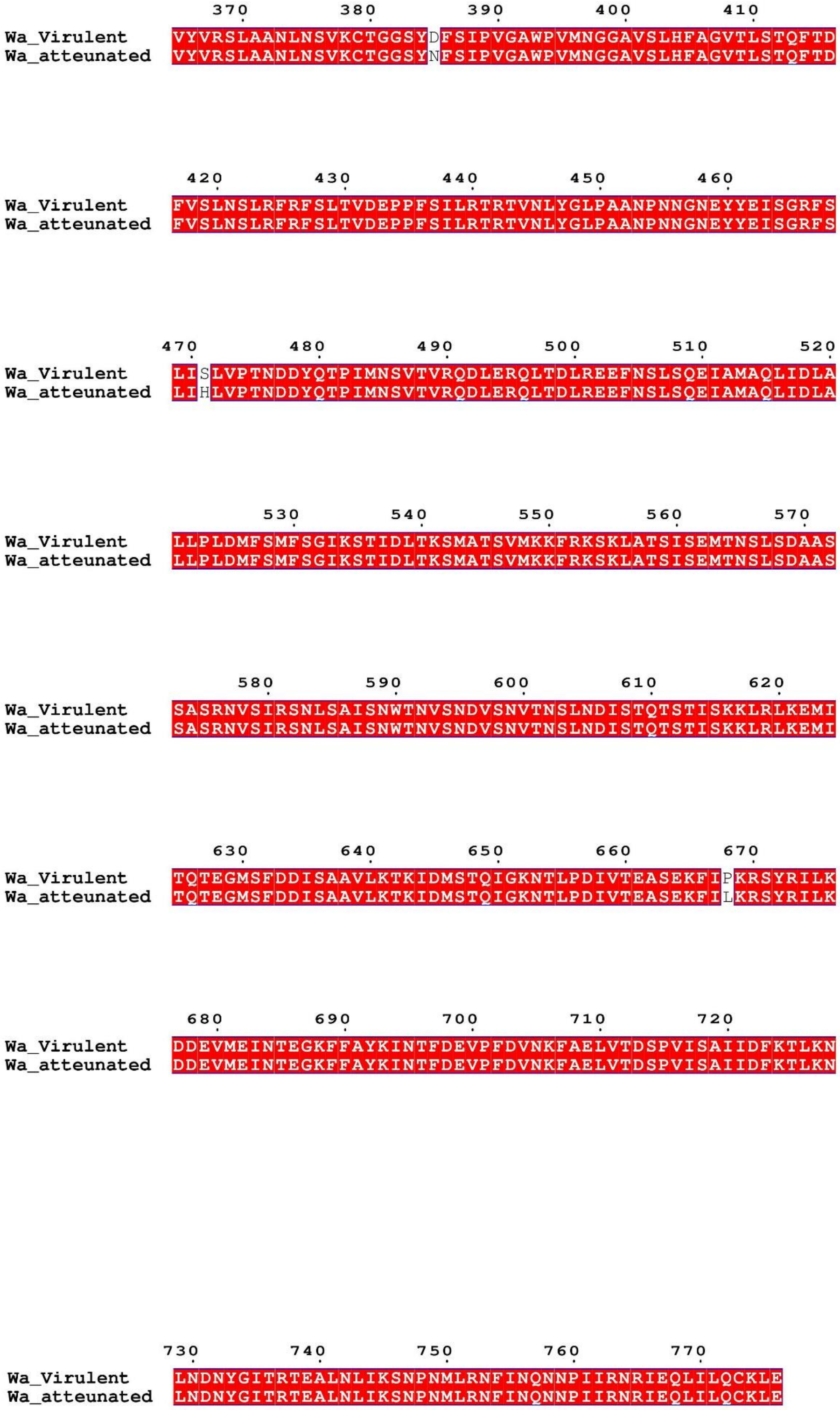

Figure S1. Amino acid alignment of VP4 gene in Wa strain.

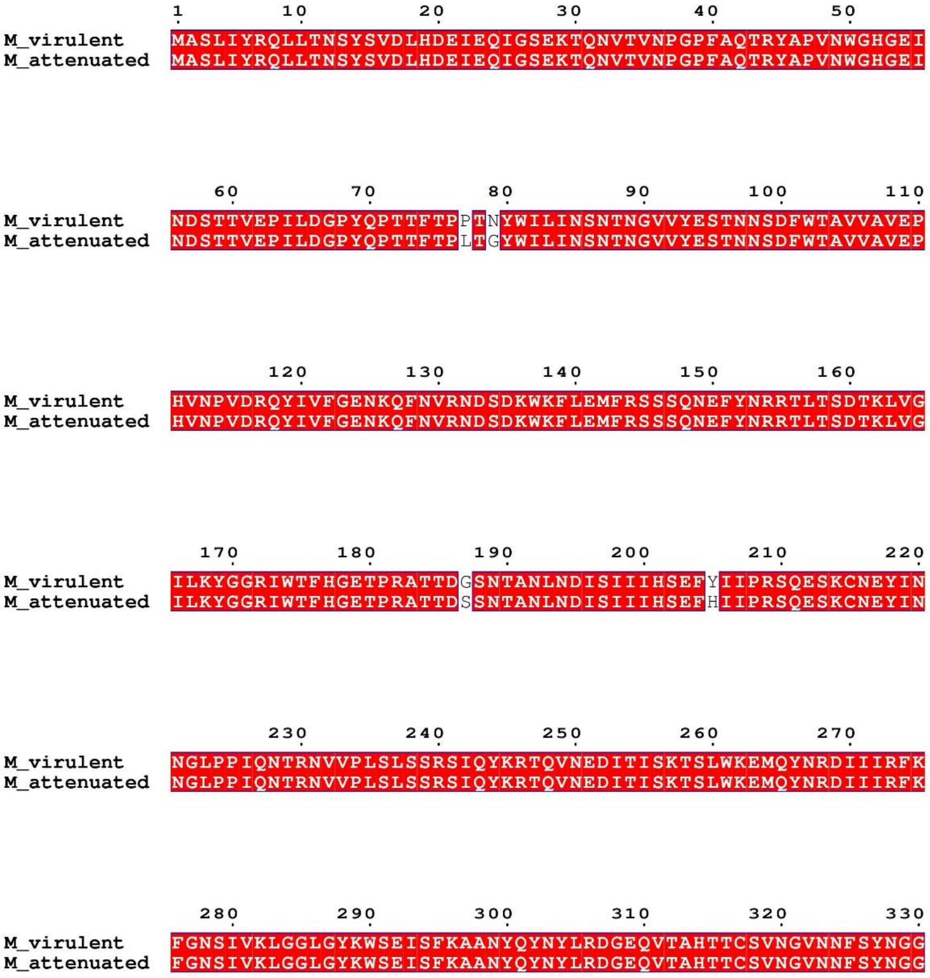
1 10 20 30 40 50
M_virulent MASLIYRQLLTNSYSVDLHDEIEQIGSEKTQNVTVNPGPFAQTRYAPVNWGHGEI
M_attenuated MASLIYRQLLTNSYSVDLHDEIEQIGSEKTQNVTVNPGPFAQTRYAPVNWGHGEI
60 70 80 90 100 110
M_virulent NDSTTVEPILDGPYQPTTFTPPTNYWILINSNTNGVVYESTNNSDFWTAVVAVEP
M_attenuated NDSTTVEPILDGPYQPTTFTPLTGYWILINSNTNGVVYESTNNSDFWTAVVAVEP
120 130 140 150 160
M_virulent HVNPVDRQYIVFGENKQFNVRNDSDKWKFLEMFRSSSQNEFYNRRTLTSDTKLVG
M_attenuated HVNPVDRQYIVFGENKQFNVRNDSDKWKFLEMFRSSSQNEFYNRRTLTSDTKLVG
170 180 190 200 210 220
M_virulent ILKYGGRIWTFHGETPRATTDGSNTANLNDISIIIHSEFYIIPRSQESKCNEYIN
M_attenuated ILKYGGRIWTFHGETPRATTDSSNTANLNDISIIIHSEFHIIPRSQESKCNEYIN
230 240 250 260 270
M_virulent NGLPPIQNTRNVVPLSLSSRSIQYKRTQVNEDITISKTSLWKEMQYNRDIIIRFK
M_attenuated NGLPPIQNTRNVVPLSLSSRSIQYKRTQVNEDITISKTSLWKEMQYNRDIIIRFK
280 290 300 310 320 330
M_virulent FGNSIVKLGGLGYKWSEISFKAANYQYNYLRDGEQVTAHTTCSVNGVNNFSYNGG
M_attenuated FGNSIVKLGGLGYKWSEISFKAANYQYNYLRDGEQVTAHTTCSVNGVNNFSYNGG

```
                 340       350       360       370       380
M_virulent     SLPTDFSISRYEVIKENSYVYVDYWDDSKAFRNMVYVRSLAANLNSVKCTGGSYD
M_attenuated   FLPTDFSISRYEVIKENSYVYVDYWDDSKAFRNMVYVRSLAANLNSVKCTGGSYN

             390       400       410       420       430       440
M_virulent     FSIPVGAWPVMNGGAVSLHFAGVTLSTQFTDFVSLNSLRFRFSLTVDEPSFSILR
M_attenuated   FSIPVGAWPVMNGGAVSLHFAGVTLSTQFTDFVSLNSLRFRFSLTVDEPSFSILR

                 450       460       470       480       490
M_virulent     TRTVNLYGLPAANPNNGNEYYEISGRFSLISLVPTNDDYQTPIMNSVTVRQDLER
M_attenuated   TRTVNLYGLPAANPNNGNEYYEISGRFSLISLVSTNDDYQTPIMNSVTVRQDLER

             500       510       520       530       540       550
M_virulent     QLTDLREEFNSLSQEIAMSQLIDLALLPLDMFSMFSGIKSTIDLTKSMATSVMKK
M_attenuated   QLTDLREEFNSLSQEIAMSQLIDLALLPLDMFSMFSGIKSTIDLTKSMATSVMKK

                 560       570       580       590       600
M_virulent     FRKSKLATSISEMTNSLSDAASSASRSVSIRSNISTISNLTNVSNDVSNVTNSLN
M_attenuated   FRKSKLATSISEMTNSLSDAASSASRSVSIRSNISTISNLTNVSNDVSNVTNSLN

             610       620       630       640       650       660
M_virulent     DISTQTSTISKKLRLREMITQTEGMSFDDISAAVLKTKIDMSTQIGKNTLPDIVT
M_attenuated   DISTQTSTISKKLRLREMITQTEGMSFDDISAAVLKTKIDMSTQIGKNTLPDIVT

                 670       680       690       700       710
M_virulent     EASEKFIPKRSYRILKDDEVMEINTEGKVFAYKIDTLNEVPFDVNKFAELVTNSP
M_attenuated   EASEKFIPKRSYRILKDDEVMEINTEGKVFAYKIDTLNEVPFDVNKFAELVTNSP

             720       730       740       750       760       770
M_virulent     VISAIIDFKTLKNLNDNYGITRIEALNLIKSNPNVLRNFINQNNPIIRNRIEQLI
M_attenuated   VISAIIDFKTLKNLNDNYGITRIEALNLIKSNPNVLRNFINQNNPIIRNRIEQLI

M_virulent     LQCKLE
M_attenuated   LQCKLE
```

Figure S2. Amino acid alignment of VP4 gene in M strain.

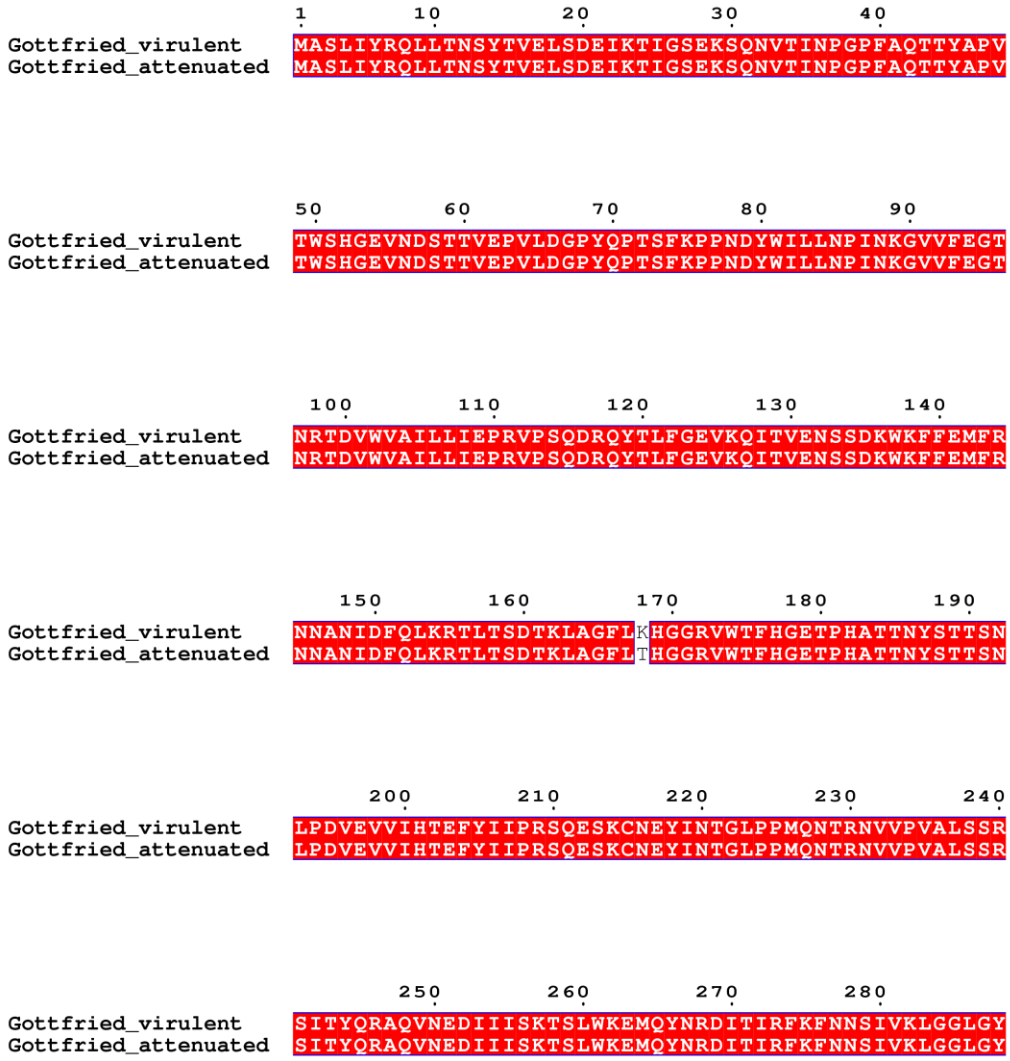
1 10 20 30 40
Gottfried_virulent MASLIYRQLLTNSYTVELSDEIKTIGSEKSQNVTINPGPFAQTTYAPV
Gottfried_attenuated MASLIYRQLLTNSYTVELSDEIKTIGSEKSQNVTINPGPFAQTTYAPV
50 60 70 80 90
Gottfried_virulent TWSHGEVNDSTTVEPVLDGPYQPTSFKPPNDYWILLNPINKGVVFEGT
Gottfried_attenuated TWSHGEVNDSTTVEPVLDGPYQPTSFKPPNDYWILLNPINKGVVFEGT
100 110 120 130 140
Gottfried_virulent NRTDVWVAILLIEPRVPSQDRQYTLFGEVKQITVENSSDKWKFFEMFR
Gottfried_attenuated NRTDVWVAILLIEPRVPSQDRQYTLFGEVKQITVENSSDKWKFFEMFR
150 160 170 180 190
Gottfried_virulent NNANIDFQLKRTLTSDTKLAGFLKHGGRVWTFHGETPHATTNYSTTSN
Gottfried_attenuated NNANIDFQLKRTLTSDTKLAGFLTHGGRVWTFHGETPHATTNYSTTSN
200 210 220 230 240
Gottfried_virulent LPDVEVVIHTEFYIIPRSQESKCNEYINTGLPPMQNTRNVVPVALSSR
Gottfried_attenuated LPDVEVVIHTEFYIIPRSQESKCNEYINTGLPPMQNTRNVVPVALSSR
250 260 270 280
Gottfried_virulent SITYQRAQVNEDIIISKTSLWKEMQYNRDITIRFKFNNSIVKLGGLGY
Gottfried_attenuated SITYQRAQVNEDIIISKTSLWKEMQYNRDITIRFKFNNSIVKLGGLGY

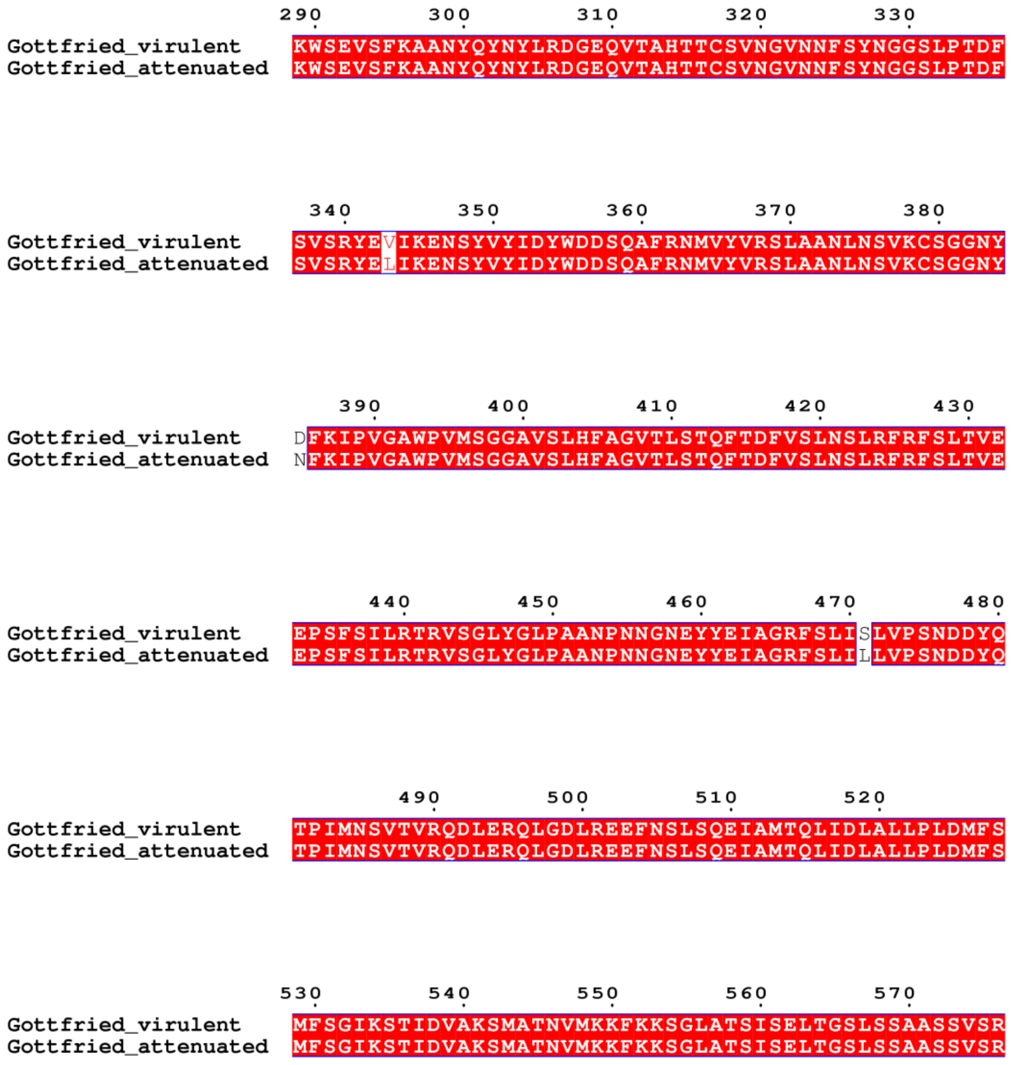
290 300 310 320 330
Gottfried_virulent KWSEVSFKAANYQYNYLRDGEQVTAHTTCSVNGVNNFSYNGGSLPTDF
Gottfried_attenuated KWSEVSFKAANYQYNYLRDGEQVTAHTTCSVNGVNNFSYNGGSLPTDF
340 350 360 370 380
Gottfried_virulent SVSRYEVIKENSYVYIDYWDDSQAFRNMVYVRSLAANLNSVKCSGGNY
Gottfried_attenuated SVSRYELIKENSYVYIDYWDDSQAFRNMVYVRSLAANLNSVKCSGGNY
390 400 410 420 430
Gottfried_virulent DFKIPVGAWPVMSGGAVSLHFAGVTLSTQFTDFVSLNSLRFRFSLTVE
Gottfried_attenuated NFKIPVGAWPVMSGGAVSLHFAGVTLSTQFTDFVSLNSLRFRFSLTVE
440 450 460 470 480
Gottfried_virulent EPSFSILRTRVSGLYGLPAANPNNGNEYYEIAGRFSLISLVPSNDDYQ
Gottfried_attenuated EPSFSILRTRVSGLYGLPAANPNNGNEYYEIAGRFSLILLVPSNDDYQ
490 500 510 520
Gottfried_virulent TPIMNSVTVRQDLERQLGDLREEFNSLSQEIAMTQLIDLALLPLDMFS
Gottfried_attenuated TPIMNSVTVRQDLERQLGDLREEFNSLSQEIAMTQLIDLALLPLDMFS
530 540 550 560 570
Gottfried_virulent MFSGIKSTIDVAKSMATNVMKKFKKSGLATSISELTGSLSSAASSVSR
Gottfried_attenuated MFSGIKSTIDVAKSMATNVMKKFKKSGLATSISELTGSLSSAASSVSR

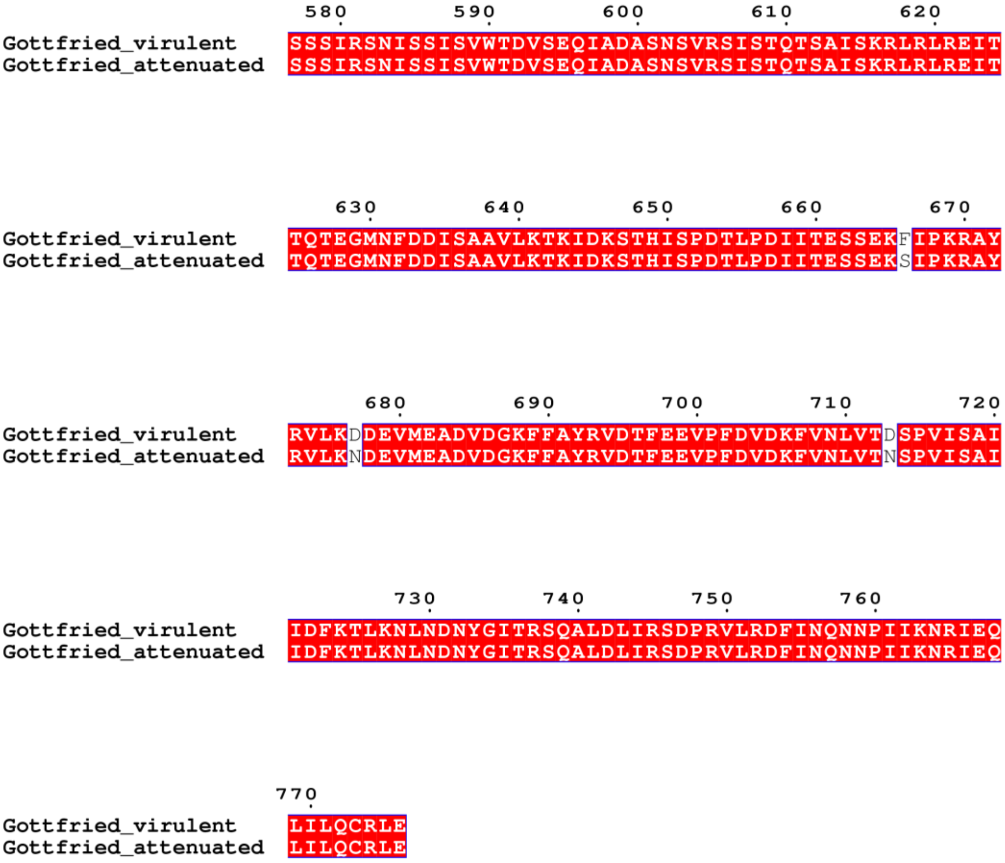

Figure S3. Amino acid alignment of VP4 gene in Gottfried strain.

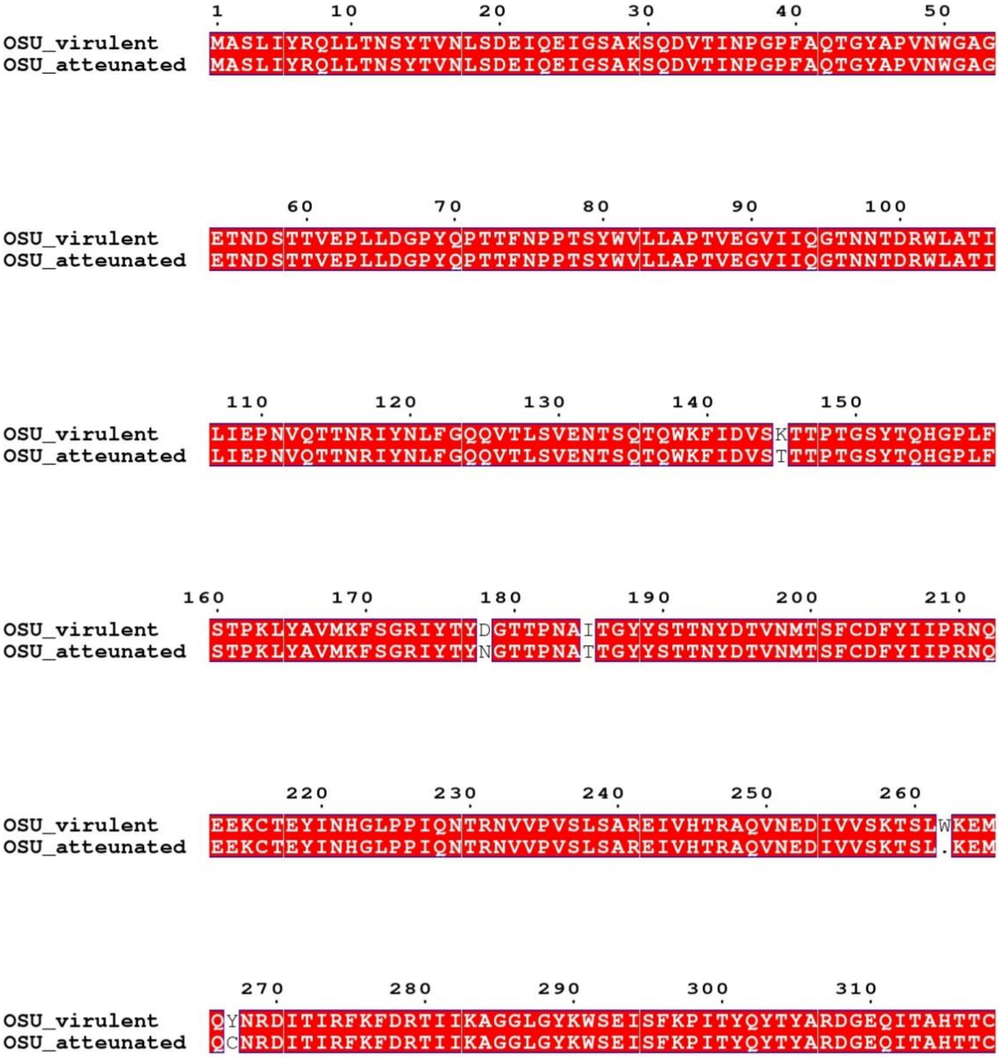
1 10 20 30 40 50
OSU_virulent MASLIYRQLLTNSYTVNLSDEIQEIGSAKSQDVTINPGPFAQTGYAPVNWGAG
OSU_atteunated MASLIYRQLLTNSYTVNLSDEIQEIGSAKSQDVTINPGPFAQTGYAPVNWGAG
60 70 80 90 100
OSU_virulent ETNDSTTVEPLLDGPYQPTTFNPPTSYWVLLAPTVEGVIIQGTNNTDRWLATI
OSU_atteunated ETNDSTTVEPLLDGPYQPTTFNPPTSYWVLLAPTVEGVIIQGTNNTDRWLATI
110 120 130 140 150
OSU_virulent LIEPNVQTTNRIYNLFGQQVTLSVENTSQTQWKFIDVSKTTPTGSYTQHGPLF
OSU_atteunated LIEPNVQTTNRIYNLFGQQVTLSVENTSQTQWKFIDVSTTTPTGSYTQHGPLF
160 170 180 190 200 210
OSU_virulent STPKLYAVMKFSGRIYTYDGTTPNAITGYYSTTNYDTVNMTSFCDFYIIPRNQ
OSU_atteunated STPKLYAVMKFSGRIYTYNGTTPNATTGYYSTTNYDTVNMTSFCDFYIIPRNQ
220 230 240 250 260
OSU_virulent EEKCTEYINHGLPPIQNTRNVVPVSLSAREIVHTRAQVNEDIVVSKTSLWKEM
OSU_atteunated EEKCTEYINHGLPPIQNTRNVVPVSLSAREIVHTRAQVNEDIVVSKTSL.KEM
270 280 290 300 310
OSU_virulent QYNRDITIRFKFDRTIIKAGGLGYKWSEISFKPITYQYTYARDGEQITAHTTC
OSU_atteunated QCNRDITIRFKFDRTIIKAGGLGYKWSEISFKPITYQYTYARDGEQITAHTTC

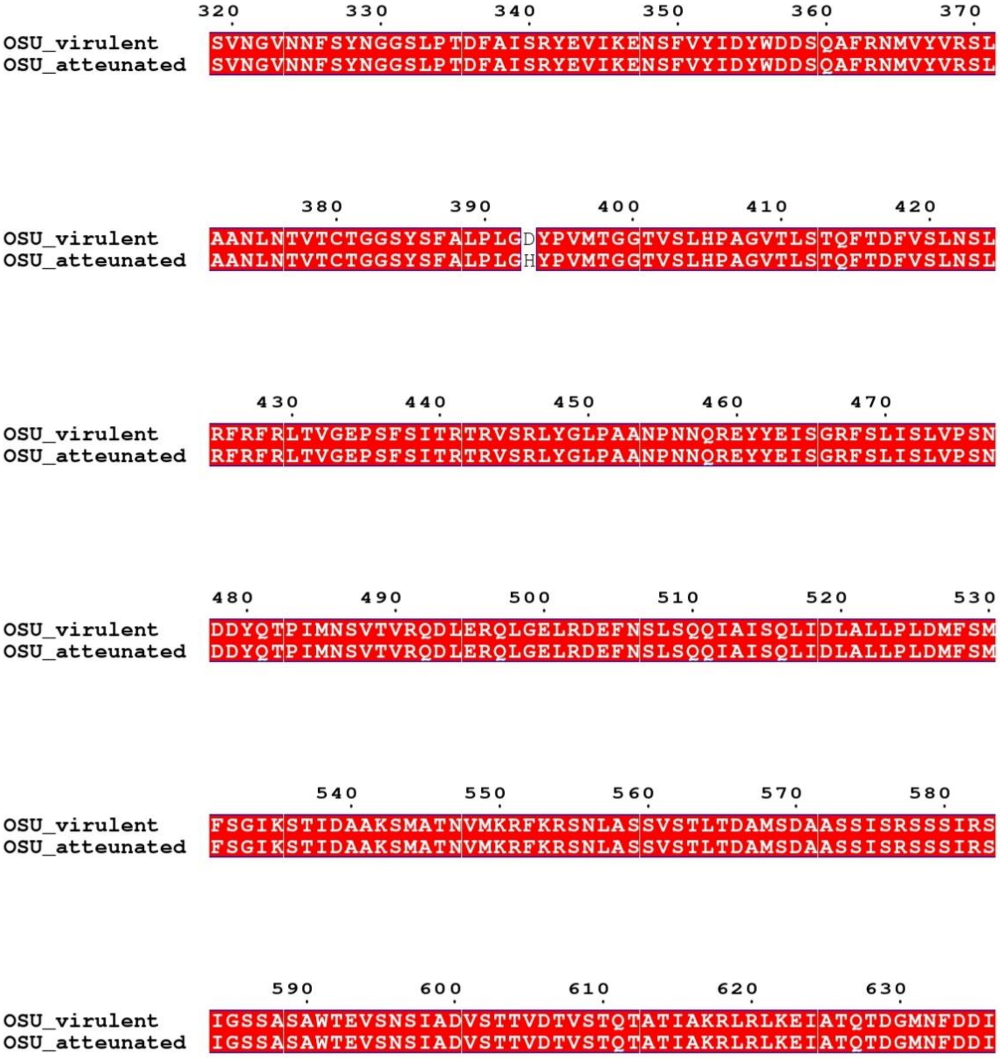

320 330 340 350 360 370
OSU_virulent SVNGVNNFSYNGGSLPTDFAISRYEVIKENSFVYIDYWDDSQAFRNMVYVRSL
OSU_atteunated SVNGVNNFSYNGGSLPTDFAISRYEVIKENSFVYIDYWDDSQAFRNMVYVRSL
380 390 400 410 420
OSU_virulent AANLNTVTCTGGSYSFALPLGDYPVMTGGTVSLHPAGVTLSTQFTDFVSLNSL
OSU_atteunated AANLNTVTCTGGSYSFALPLGHYPVMTGGTVSLHPAGVTLSTQFTDFVSLNSL
430 440 450 460 470
OSU_virulent RFRFRLTVGEPSFSITRTRVSRLYGLPAANPNNQREYYEISGRFSLISLVPSN
OSU_atteunated RFRFRLTVGEPSFSITRTRVSRLYGLPAANPNNQREYYEISGRFSLISLVPSN
480 490 500 510 520 530
OSU_virulent DDYQTPIMNSVTVRQDLERQLGELRDEFNSLSQQIAISQLIDLALLPLDMFSM
OSU_atteunated DDYQTPIMNSVTVRQDLERQLGELRDEFNSLSQQIAISQLIDLALLPLDMFSM
540 550 560 570 580
OSU_virulent FSGIKSTIDAAKSMATNVMKRFKRSNLASSVSTLTDAMSDAASSISRSSSIRS
OSU_atteunated FSGIKSTIDAAKSMATNVMKRFKRSNLASSVSTLTDAMSDAASSISRSSSIRS
590 600 610 620 630
OSU_virulent IGSSASAWTEVSNSIADVSTTVDTVSTQTATIAKRLRLKEIATQTDGMNFDDI
OSU_atteunated IGSSASAWTEVSNSIADVSTTVDTVSTQTATIAKRLRLKEIATQTDGMNFDDI

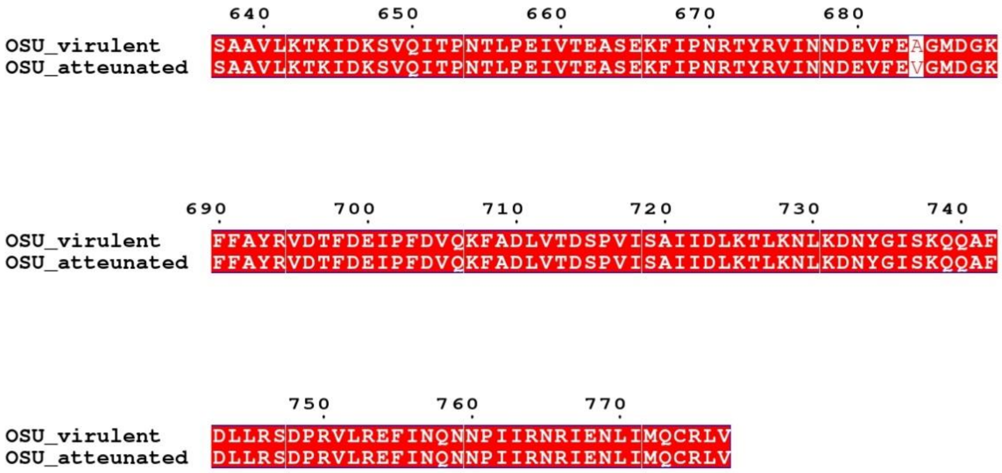

Figure S4. Amino acid alignment of VP4 gene in OSU strain.

Table S1. NCBI accession number of all gene segments.

strain	gene	Virulent/Attenuated	NCBI Accession Number
Wa	NSP1	Virulent	MT025852
Wa	NSP1	attenuated	MT025853
Wa	NSP2	Virulent	MT025854
Wa	NSP2	attenuated	MT025855
Wa	NSP3	Virulent	MT025856
Wa	NSP3	attenuated	MT025857
Wa	NSP4	Virulent	MT025858
Wa	NSP4	attenuated	MT025859
Wa	NSP5	Virulent	MT025860
Wa	NSP5	attenuated	MT025861
Wa	VP1	Virulent	MT025862
Wa	VP1	attenuated	MT025863
Wa	VP2	Virulent	MT025864
Wa	VP2	attenuated	MT025865
Wa	VP3	Virulent	MT025866
Wa	VP3	attenuated	MT025867
Wa	VP4	Virulent	MT025868
Wa	VP4	attenuated	MT025869
Wa	VP6	Virulent	MT025870
Wa	VP6	attenuated	MT025871
Wa	VP7	Virulent	MT025872
Wa	VP7	attenuated	MT025873
M	NSP1	Virulent	MT025874
M	NSP1	attenuated	MT025875
M	NSP2	Virulent	MT025876
M	NSP2	attenuated	MT025877
M	NSP3	Virulent	MT025878
M	NSP3	attenuated	MT025879
M	NSP4	Virulent	MT025880
M	NSP4	attenuated	MT025881
M	NSP5	Virulent	MT025882
M	NSP5	attenuated	MT025883
M	VP1	Virulent	MT025884

M	VP1	attenuated	MT025885
M	VP2	Virulent	MT025886
M	VP2	attenuated	MT025887
M	VP3	Virulent	MT025888
M	VP3	attenuated	MT025889
M	VP4	Virulent	MT025890
M	VP4	attenuated	MT025891
M	VP6	Virulent	MT025892
M	VP6	attenuated	MT025893
M	VP7	Virulent	MT025894
M	VP7	attenuated	MT025895
Gottfried	NSP1	Virulent	MT025896
Gottfried	NSP1	attenuated	MT025897
Gottfried	NSP2	Virulent	MT025898
Gottfried	NSP2	attenuated	MT025899
Gottfried	NSP3	Virulent	MT025900
Gottfried	NSP3	attenuated	MT025901
Gottfried	NSP4	Virulent	MT025902
Gottfried	NSP4	attenuated	MT025903
Gottfried	NSP5	Virulent	MT025904
Gottfried	NSP5	attenuated	MT025905
Gottfried	VP1	Virulent	MT025906
Gottfried	VP1	attenuated	MT025907
Gottfried	VP2	Virulent	MT025908
Gottfried	VP2	attenuated	MT025909
Gottfried	VP3	Virulent	MT025910
Gottfried	VP3	attenuated	MT025911
Gottfried	VP4	Virulent	MT025912
Gottfried	VP4	attenuated	MT025913
Gottfried	VP6	Virulent	MT025914
Gottfried	VP6	attenuated	MT025915
Gottfried	VP7	Virulent	MT025916
Gottfried	VP7	attenuated	MT025917
OSU	NSP1	Virulent	MT025918
OSU	NSP1	attenuated	MT025919
OSU	NSP2	Virulent	MT025920
OSU	NSP2	attenuated	MT025921
OSU	NSP3	Virulent	MT025922
OSU	NSP3	attenuated	MT025923
OSU	NSP4	Virulent	MT025924
OSU	NSP4	attenuated	MT025925
OSU	NSP5	Virulent	MT025926
OSU	NSP5	attenuated	MT025927
OSU	VP1	Virulent	MT025928
OSU	VP1	attenuated	MT025929
OSU	VP2	Virulent	MT025930
OSU	VP2	attenuated	MT025931
OSU	VP3	Virulent	MT025932
OSU	VP3	attenuated	MT025933
OSU	VP4	Virulent	MT025934
OSU	VP4	attenuated	MT025935
OSU	VP6	Virulent	MT025936
OSU	VP6	attenuated	MT025937
OSU	VP7	Virulent	MT025938
OSU	VP7	attenuated	MT025939

Table S2. dN/dS ratio analyze per site in the region 500-660 of VP4 gene in Wa and M strains.

Codon#	Codon Start	Triplet	Syn (s)	Nonsyn (n)	Syn sites (S)	Nonsyn sites (N)	dS	dN	dN-dS	P-value	Normalized dN-dS
524	1570	CTA	2	0	0.991138	1.689134	2.017882	0	−2.01788	1	−20.43527309
539	1615	TTA	2	0	1.052584	1.728064	1.900086	0	−1.90009	1	−19.24234022
587	1759	ATT	1	0	0.802364	2.197636	1.246318	0	−1.24632	1	−12.62156956
657	1969	GAC	1	0	0.363339	2.636661	2.752249	0	−2.75225	1	−27.87226884

dN/dS ratios calculation per site was performed directly using the MEGA7 software. Codon# denotes the aa position within the VP4 gene. The normalized dN-dS denotes results. These results indicate there were strong negative selection in the region 500–660 of VP4 gene in Wa and M strains.
